# MUTYH is associated with hepatocarcinogenesis in a non-alcoholic steatohepatitis mouse model

**DOI:** 10.1038/s41598-021-83138-8

**Published:** 2021-02-11

**Authors:** Hiroki Sakamoto, Koji Miyanishi, Shingo Tanaka, Ryo Ito, Kota Hamaguchi, Akira Sakurada, Masanori Sato, Tomohiro Kubo, Takahiro Osuga, Kazuyuki Murase, Kohichi Takada, Yusaku Nakabeppu, Masayoshi Kobune, Junji Kato

**Affiliations:** 1 Department of Medical Oncology, Sapporo Medical University School of Medicine, South-1, West-16, Chuo-ku, Sapporo, 060-8543 Japan; 2 Department of Hematology, Sapporo Medical University School of Medicine, South-1, West-16, Chuo-ku, Sapporo, 060-8543 Japan; 3 Division of Neurofunctional Genomics, Department of Immunobiology and Neuroscience, Medical Institute of Bioregulation, Kyushu University, 3-1-1 Maidashi, Higashi-Ku, Fukuoka, 812-8582 Japan

**Keywords:** Cancer, Molecular biology, Biomarkers, Diseases, Gastroenterology, Oncology, Risk factors

## Abstract

Non-alcoholic steatohepatitis (NASH)-related HCC is associated with oxidative stress. However, the mechanisms underlying the development of NASH-related HCC is unclear. MUTYH is one of the enzymes that is involved in repair of oxidative DNA damage. The aim of this study was to investigate the association between MUTYH and NASH-related hepatocarcinogenesis. MUTYH wild-type (*Mutyh*^+/+^), heterozygous (*Mutyh*^+/−^), and MUTYH-null (*Mutyh*^−/−^) mice were fed a high-fat high-cholesterol (HFHC) diet or HFHC + high iron diet (20 mice per group) for 9 months. Five of 20 *Mutyh*^−/−^ mice fed an HFHC + high iron diet developed liver tumors, and they developed more liver tumors than other groups (especially vs. Mutyh^+/+^ fed an HFHC diet, *P* = 0.0168). Immunohistochemical analysis revealed significantly higher accumulation of oxidative stress markers in mice fed an HFHC + high iron diet. The gene expression profiles in the non-tumorous hepatic tissues were compared between wild-type mice that developed no liver tumors and MUTYH-null mice that developed liver tumors. Gene Set Enrichment Analysis identified the involvement of the Wnt/β-catenin signaling pathway and increased expression of *c-Myc* in MUTYH-null liver. These findings suggest that MUTYH deficiency is associated with hepatocarcinogenesis in patients with NASH with hepatic iron accumulation.

## Introduction

Non-alcoholic fatty liver disease (NAFLD) is a chronic hepatic disease with an increasing incidence worldwide and which affects approximately 25% of all adults^1^. NAFLD has been reported to be related to the increased incidence of lifestyle diseases, including diabetes mellitus and dyslipidemia^2^. Although NAFLD is simple steatosis in most cases, about 10% of patients develop non-alcoholic steatohepatitis (NASH)^3^. Ascha et al. reported that the annual incidence of hepatocellular carcinoma (HCC) was 2.6% in patients with NASH cirrhosis and 4.0% in hepatitis C virus (HCV) cirrhosis. Thus, the incidence of NASH-related HCC seems comparable to that of HCV-related HCC^4^.

Oxidative stress caused by excessive iron has been shown to be one of the risk factors for the development of various cancers^5^. There is organ-specificity in carcinogenesis by oxidative stress, such as hepatocarcinogenesis in the liver affected by hereditary hemochromatosis. Regarding role of iron storage in the liver, we have reported that excessive iron is produced in the livers of patients with chronic hepatitis C and HCV-related hepatocarcinogenesis is strongly associated with oxidative stress^6^. It has been reported that NASH-related HCC is associated with oxidative stress^7,8^. However, the mechanisms underlying the development of NASH-related HCC remain unclear.

It has been reported that the hepatic iron concentration is increased in the liver affected by NASH^9,10^. Although it is not substantiated by the literature that higher levels of intrahepatic iron are associated with a higher HCC risk in NASH patients, increased hepatic iron facilitates the formation of reactive oxygen species (ROS), including the hydroxyl radical. 8-oxo-7,8-dihydroguanine (8-oxoguanine or 8-oxoG) is a marker of oxidative DNA damage; it is produced by oxidation of the C8 of guanine and causes a G:C to T:A transversion through its ability to pair with both adenine and cytosine^11,12^. Oxidative DNA damage can lead to tumor formation when it is generated within genes involved in carcinogenesis, including oncogenes and tumor suppressor genes. *MTH1*, *OGG1* and *MUTYH* genes encode enzymes required to maintain low levels of 8-oxoG-induced mutagenesis. MTH1 hydrolyzes 8-oxo-7,8-dihydro-2-deoxyguanosine triphosphate (8-oxo-dGTP), an oxidized form of dGTP in the nucleotide pool, to 8-oxo-dGMP and pyrophosphate, and OGG1 excises 8-oxo-G opposite cytosine in DNA thus avoiding its accumulation in DNA. MUTYH excises adenine which pairs with 8-oxoG, thus efficiently suppresses 8-oxoG-induced mutagenesis^13^.

Biallelic MUTYH mutations are found in 0.01–0.04% of the Caucasian population and are associated with an extremely high risk of developing colorectal adenomas and cancer^14–17^. For individuals with a monoallelic mutation, the risk is considered to be moderately increased^14,15,17^. There are only a few studies that investigated liver tumors in the context of biallelic or monoallelic MUTYH mutations^18–21^. However, the significance of MUTYH deficiency in hepatocarcinogenesis in patients with NASH is unclear.

The aim of the present study was to examine the relationship between MUTYH deficiency and hepatocarcinogenesis in patients with NASH. For this purpose, we established a murine NASH model by feeding a high-fat high-carbohydrate (HFHC) diet and high iron diet to investigate the incidence of liver tumors in MUTYH-null and wild-type mice and to carry out gene expression profiling in non-tumorous hepatic tissues from MUTYH-null mice with the development of liver tumors and wild-type mice without the development of liver tumors. We further examined the possibility of preventing hepatocarcinogenesis in patients with NASH using antioxidants.

## Results

### Comparison of body weights, serum alanine aminotransferase levels, and hepatic iron concentrations in the mice

**Figure 1:**
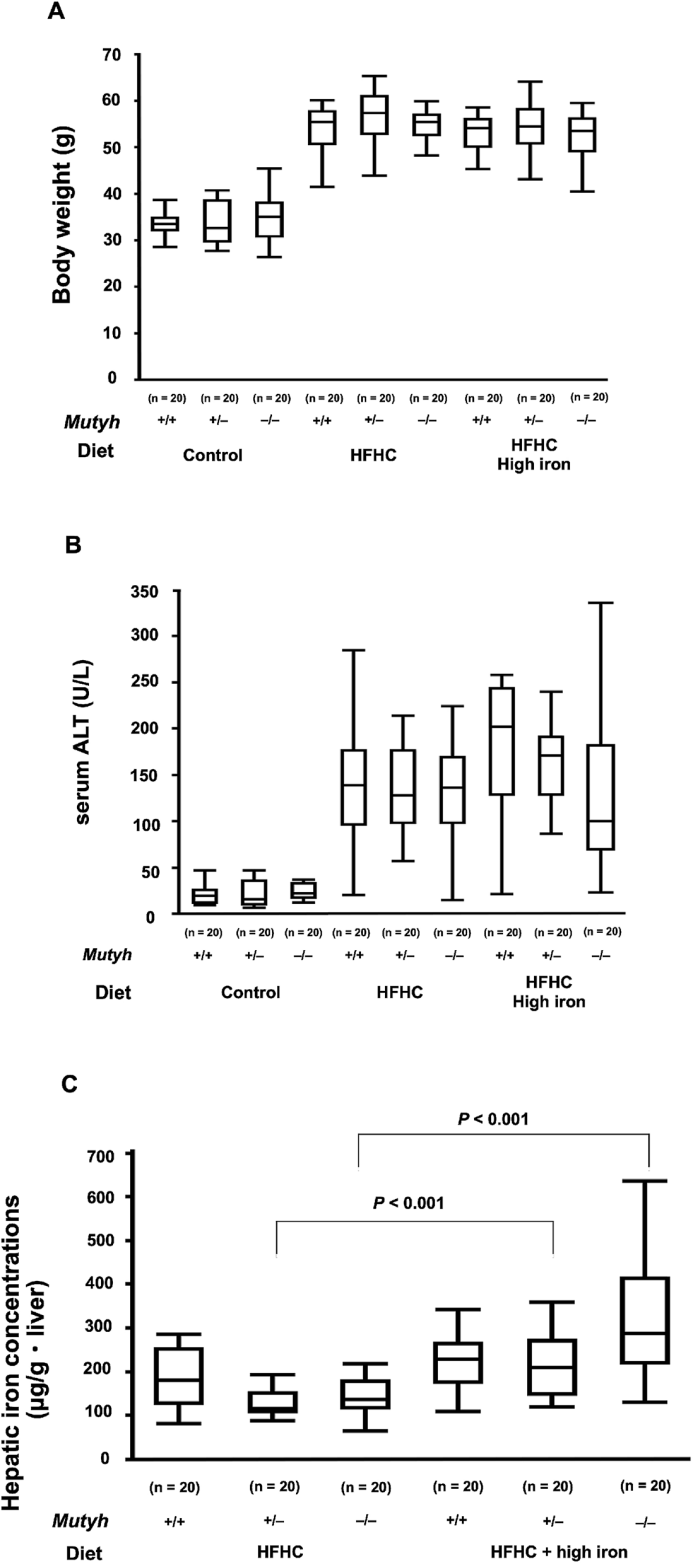
(**A**) Body weights, (**B**) serum ALT and (**C**) hepatic iron concentrations. Body weights (**A**) and serum ALT (**B**) are significantly higher in the groups fed with an HFHC diet (the HFHC diet group and the HFHC + high iron diet group) than the control group (**A**): *Mutyh*^+/+^, HFHC diet group vs. *Mutyh*^+/+^, control diet group; *P* < 0.001, *Mutyh*^+/−^, HFHC diet group vs. *Mutyh*^+/−^, control diet group ; *P* < 0.001, *Mutyh*^−/−^, HFHC diet group vs. *Mutyh*^−/−^, control diet group; *P* = 0.002, *Mutyh*^+/+^, HFHC + high iron diet group vs. *Mutyh*^+/+^, HFHC diet group; *P* = 0.0925, MUTYH^+/−^, HFHC + high iron diet group vs. *Mutyh*^+/−^, HFHC diet group; *P* = 0.5256, *Mutyh*^−/−^, HFHC + high iron diet group vs. *Mutyh*^−/−^, HFHC diet group; *P* = 0.8564) (**B**): *Mutyh*^+/+^, HFHC diet group vs. *Mutyh*^+/+^, control diet group; *P* < 0.001, *Mutyh*^+/−^, HFHC diet group vs. *Mutyh*^+/−^, control diet group ; *P* = 0.0082, *Mutyh*^−/−^, HFHC diet group vs. *Mutyh*^−/−^, control diet group ; *P* < 0.001, *Mutyh*^+/+^, HFHC + high iron diet group vs. *Mutyh*^+/+^, HFHC diet group ; *P* = 0.0232, *Mutyh*^+/−^, HFHC + high iron diet group vs. *Mutyh*^+/−^, HFHC diet group ; *P* = 0.0613, *Mutyh*^−/−^, HFHC + high iron diet group vs. *Mutyh*^−/−^, HFHC diet group ; *P* = 0.7415). Hepatic iron concentrations (**C**) are significantly higher in the high iron diet groups of mice with genotypes of *Mutyh*^+/−^ and *Mutyh*^−/−^ (*Mutyh*^+/+^, HFHC + high iron diet group vs. *Mutyh*^+/+^, HFHC diet group; *P* = 0.3359, *Mutyh*^+/−^, HFHC + high iron diet group vs. *Mutyh*^+/−^, HFHC diet group; *P* < 0.001, *Mutyh*^−/−^, HFHC + high iron diet group vs. *Mutyh*^−/−^, HFHC diet group; *P* < 0.0001). Data are shown as box plots for each group of mice. Median values are shown by the line within the box. The bottom and top edges of the boxes represent the 25th and 75th percentiles, respectively. ALT, alanine aminotransferase; HFHC, high-fat high-carbohydrate.

Compared to the control group, body weights and serum alanine aminotransferase (ALT) levels significantly increased in the HFHC diet group and HFHC + high iron diet group (Fig. 1A,B). Hepatic iron concentrations significantly increased in the high iron diet groups of both the *Mutyh*^+/−^ and *Mutyh*^−/−^ genotypes (Fig. 1C). The hepatic iron concentration in the HFHC diet group was similar to that in the mice fed the control diet in our previous report^14^, suggesting that the increased hepatic iron concentration may result from feeding with a high iron diet.

### Histological findings of the livers (including the degree of hepatic fibrosis) of the mice of each group

**Figure 2:**
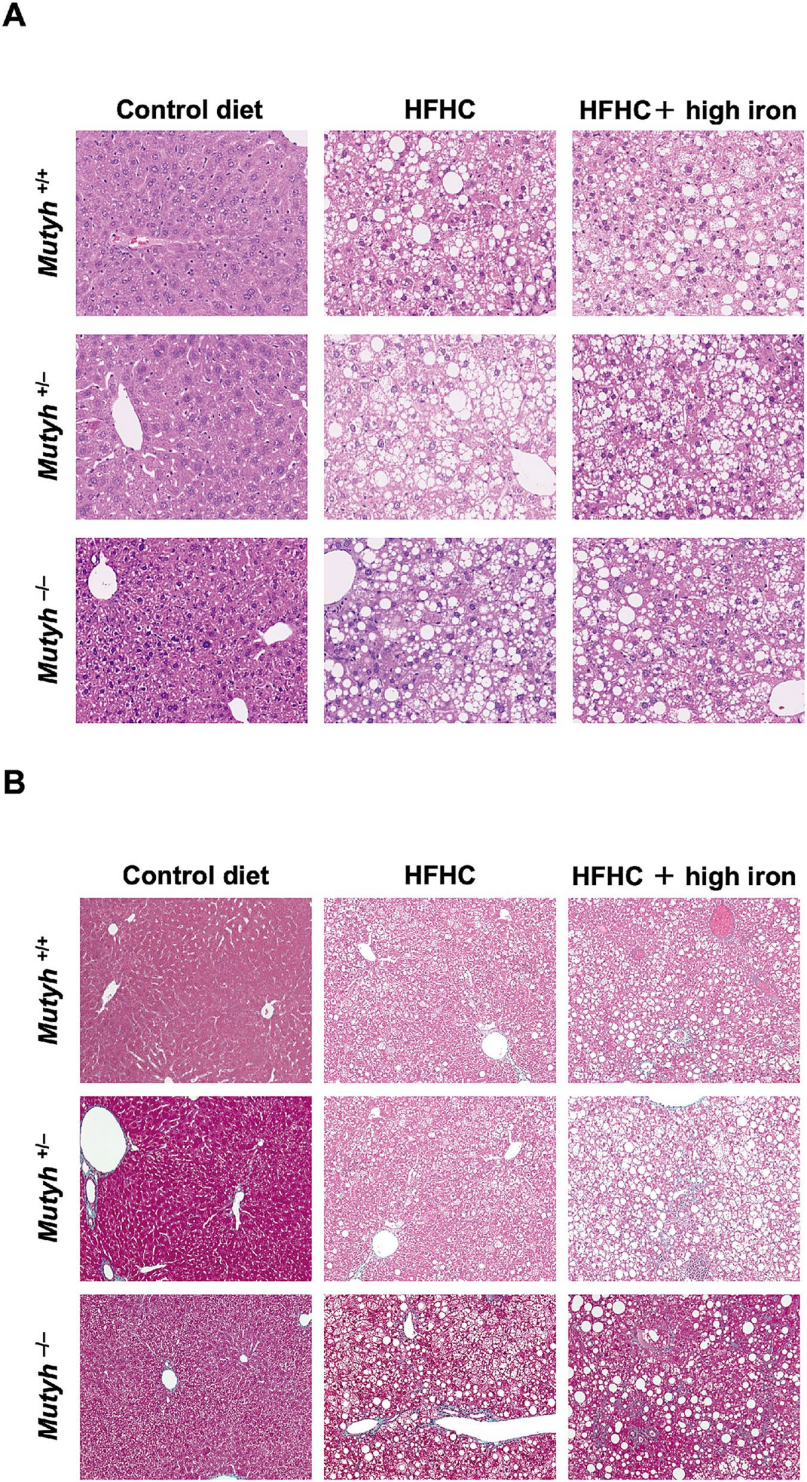
(**A**) Liver section (H&E × 100). In the groups fed an HFHC diet, prominent hepatic steatosis is seen associated with inflammatory infiltration and hepatocyte ballooning, regardless of a high-iron diet. No difference was seen between the genotypes. (**B**) Liver section (Masson trichrome stain × 100). In groups fed an HFHC diet, mild fibrosis was seen around the central veins, portal veins, and hepatocytes. HFHC, high-fat high-carbohydrate; H&E, hematoxylin & eosin.

The histological features of the livers of the groups fed with an HFHC diet were characterized by prominent hepatic steatosis, associated with inflammatory infiltration and hepatocyte ballooning, regardless of a high iron diet. There was no difference between *Mutyh* genotypes (Fig. 2A). Evaluation of hepatic fibrosis by MT staining showed mild fibrosis around the central veins, portal veins, and hepatocytes (Fig. 2B). Scores for grading and staging of NASH are shown in Supplementary Table 1.

Steatosis grade, lobular inflammation, and fibrosis stage were significantly higher in mice fed an HFHC diet or HFHC + high iron diet than those fed a control diet. There were no differences between an HFHC diet and HFHC + high iron diet, irrespective of genotype. In the mice fed an HFHC + high iron diet, intrahepatic iron deposition was observed in both the parenchyma and interstitium.

### The development of hepatic tumors in each group

**Figure 3:**
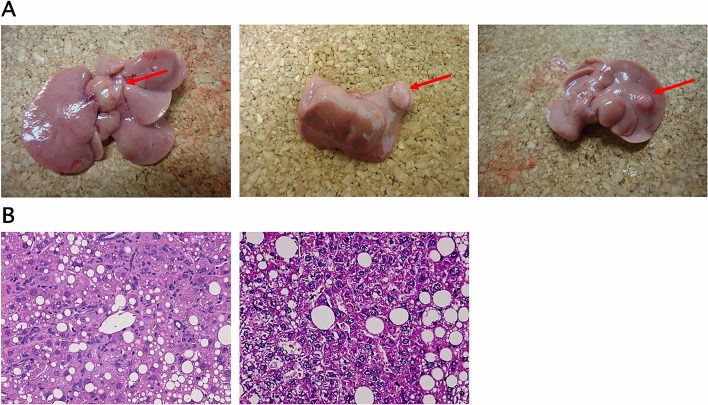
*Mutyh*^−/−^ mice fed an HFHC + high iron diet developed liver tumors. (**A**) Arrows indicate liver tumors. Gross liver appearance of *Mutyh*^−/−^ mice fed an HFHC + high iron diet, showing single tumor foci at the time of necropsy. (**B**) Liver tumor sections (H&E × 100). HFHC, high-fat high-carbohydrate; H&E, hematoxylin & eosin.

The liver tumors which developed were whitish and solitary, with tumor cells with increased nucleus to cytoplasm ratios and an irregular arrangement of the hepatic cords, as observed with HE staining. Liver tumor morphology was compatible with HCC (Fig. 3). Liver tumors developed in 25% of the *Mutyh*^−/−^ HFHC + high iron diet group. The incidence of liver tumors was significantly higher in the *Mutyh*^−/−^ HFHC + high iron diet group than the *Mutyh*^+/+^ HFHC diet group or the *Mutyh*^−/−^ HFHC diet group (*Mutyh*^−/−^ HFHC + high iron diet group vs *Mutyh*^+/+^ HFHC diet group *P* = 0.0168) (Table 1). Administration of N-acetyl L-cysteine (NAC), an antioxidant known to act directly and/or by increasing intracellular GSH38, to this group (*Mutyh*^−/−^ HFHC + high iron diet group) resulted in a reduction of the incidence of liver tumors by almost half. Only one MUTYH^+/+^ mouse fed an HFHC diet developed an extrahepatic tumor, this being in the small intestine.

**Table 1:** Liver tumor incidence.

Feeding period	Diet	*Mutyh* Genotype	Numbers of mice	
			Total	Liver tumor
9 months	HFHC	*Mutyh*^+/+^	20	0 (0%)*
	HFHC	*Mutyh*^+/−^	20	2 (10%)
	HFHC	*Mutyh*^−/−^	20	1 (5%)
	HFHC + high iron	*Mutyh*^+/+^	20	0 (0%)
	HFHC + high iron	*Mutyh*^+/−^	20	2 (10%)
	HFHC + high iron	*Mutyh*^−/−^	20	5 (25%)*
	HFHC + high iron + NAC	*Mutyh*^−/−^	15	2 (13%)

*Chi-square test (HFHC + high iron diet *Mutyh*^−/−^ group vs HFHC *Mutyh*^+/+^ group) *P* = 0.0168.

HFHC, high-fat high-carbohydrate; NAC, N-acetyl L-cysteine.

### Immunostaining with anti-4-HNE antibody and semiquantitative analysis

**Figure 4:**
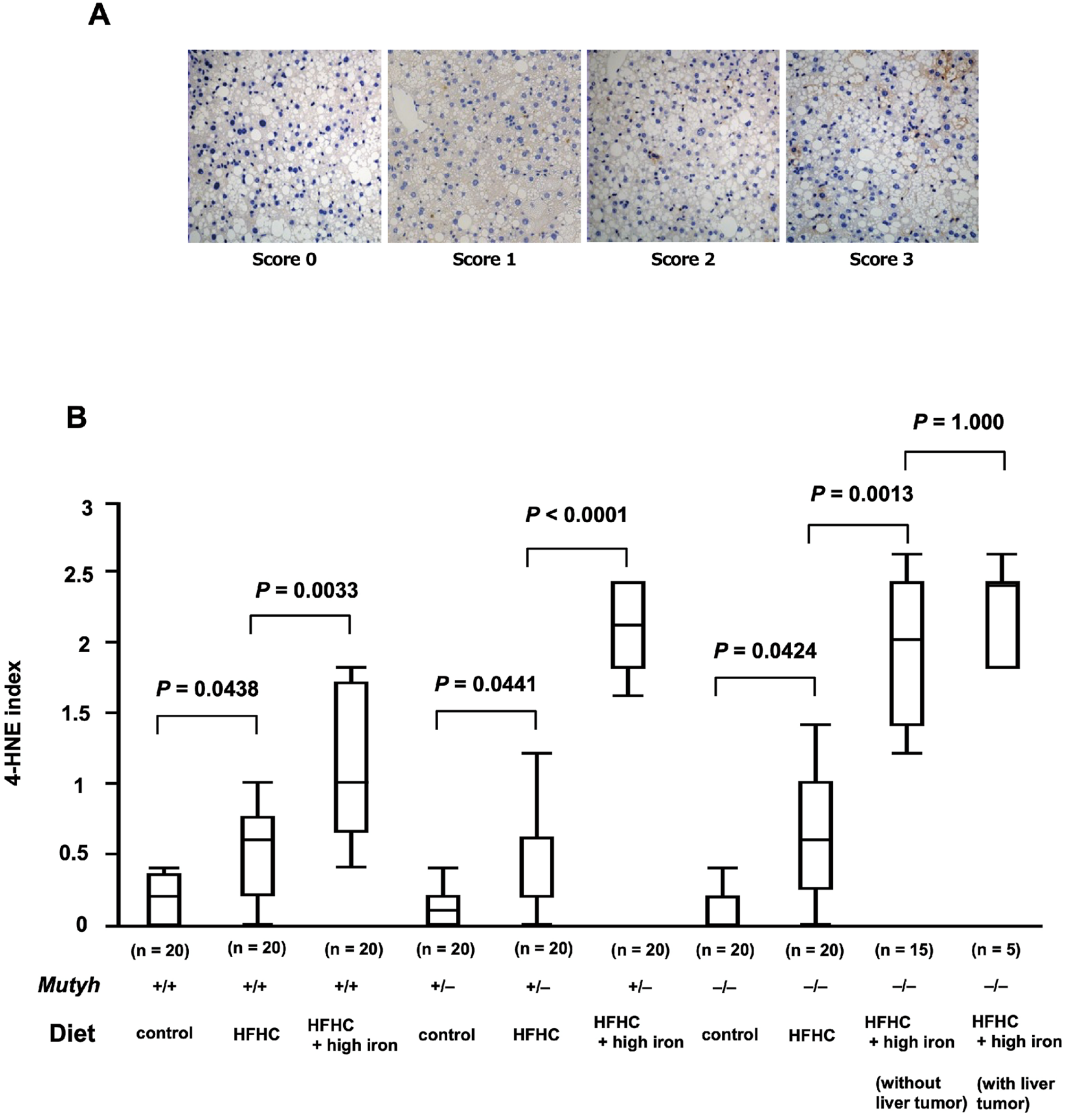
Liver sections stained with 4-HNE stain. (**A**) The intensity of 4-HNE immunostaining was scored from 0 to 3; 0 no staining, 1 mild (punctuated labeling), 2 moderate (dense labeling in a few cells), 3 strong (dense and homogenous labeling in numerous cells). At least five random fields were examined for each sample and the average of the scores was determined as the 4-HNE index. (**B**) The 4-HNE stain index. The index was significantly higher in the HFHC diet groups than the control diet group (*Mutyh*^+/+^, HFHC diet group vs. *Mutyh*^+/+^, control diet group; *P* = 0.438, *Mutyh*^+/−^, HFHC diet group vs. *Mutyh*^+/−^, control diet group; *P* = 0.0441, *Mutyh*^−/−^, HFHC diet group vs. *Mutyh*^−/−^, control diet group; *P* = 0.0424) and significantly higher in the HFHC + high-iron diet groups than the HFHC diet groups (*Mutyh*^+/+^, HFHC + high-iron diet group vs. *Mutyh*^+/+^, HFHC diet group; *P* = 0.0033, *Mutyh*^+/−^, HFHC + high-iron diet group vs. *Mutyh*^+/−^, HFHC diet group; *P* < 0.0001, *Mutyh*^−/−^, HFHC + high-iron diet group vs. *Mutyh*^−/−^, HFHC diet group; *P* = 0.0013). There was no significant difference in the index according to the presence or absence of tumor development (*P* = 1.000). Data analysed with a Kruskal–Wallis test followed by Dunn–Bonferroni test. For all data analysis *P* < 0.05 considered significant. Data are shown as box plots for each group of mice. Median values are shown by the line within the box. The bottom and top edges of the boxes represent the 25th and 75th percentiles, respectively. HFHC, high-fat high-carbohydrate; 4-HNE, 4-hydroxynonenal.

The 4-HNE index, determined by anti-4-HNE immunostaining, was used to evaluate lipid peroxidation (Fig. 4A). For each genotype, the index was significantly higher in the HFHC diet groups than the control diet group (*Mutyh*^+/+^, HFHC diet group vs. *Mutyh*^+/+^, control diet group; *P* = 0.438, *Mutyh*^+/−^, HFHC diet group vs. *Mutyh*^+/−^, control diet group; *P* = 0.0441, *Mutyh*^−/−^, HFHC diet group vs. *Mutyh*^−/−^, control diet group; *P* = 0.0424) and significantly higher in the HFHC + high-iron diet groups than the HFHC diet groups (*Mutyh*^+/+^, HFHC + high-iron diet group vs. *Mutyh*^+/+^, HFHC diet group; *P* = 0.0033, *Mutyh*^+/−^, HFHC + high-iron diet group vs. *Mutyh*^+/−^, HFHC diet group; *P* < 0.0001, *Mutyh*^−/−^, HFHC + high-iron diet group vs. *Mutyh*^−/−^, HFHC diet group; *P* = 0.0013). There was no significant difference in the index according to the presence or absence of tumor development (*P* = 1.000) (Fig. 4B). These findings suggest that hepatic lipid peroxidation increased with a high iron diet, regardless of the *Mutyh* genotype.

### Immunostaining with anti-8-oxo-dG antibody and semiquantitative analysis

**Figure 5:**
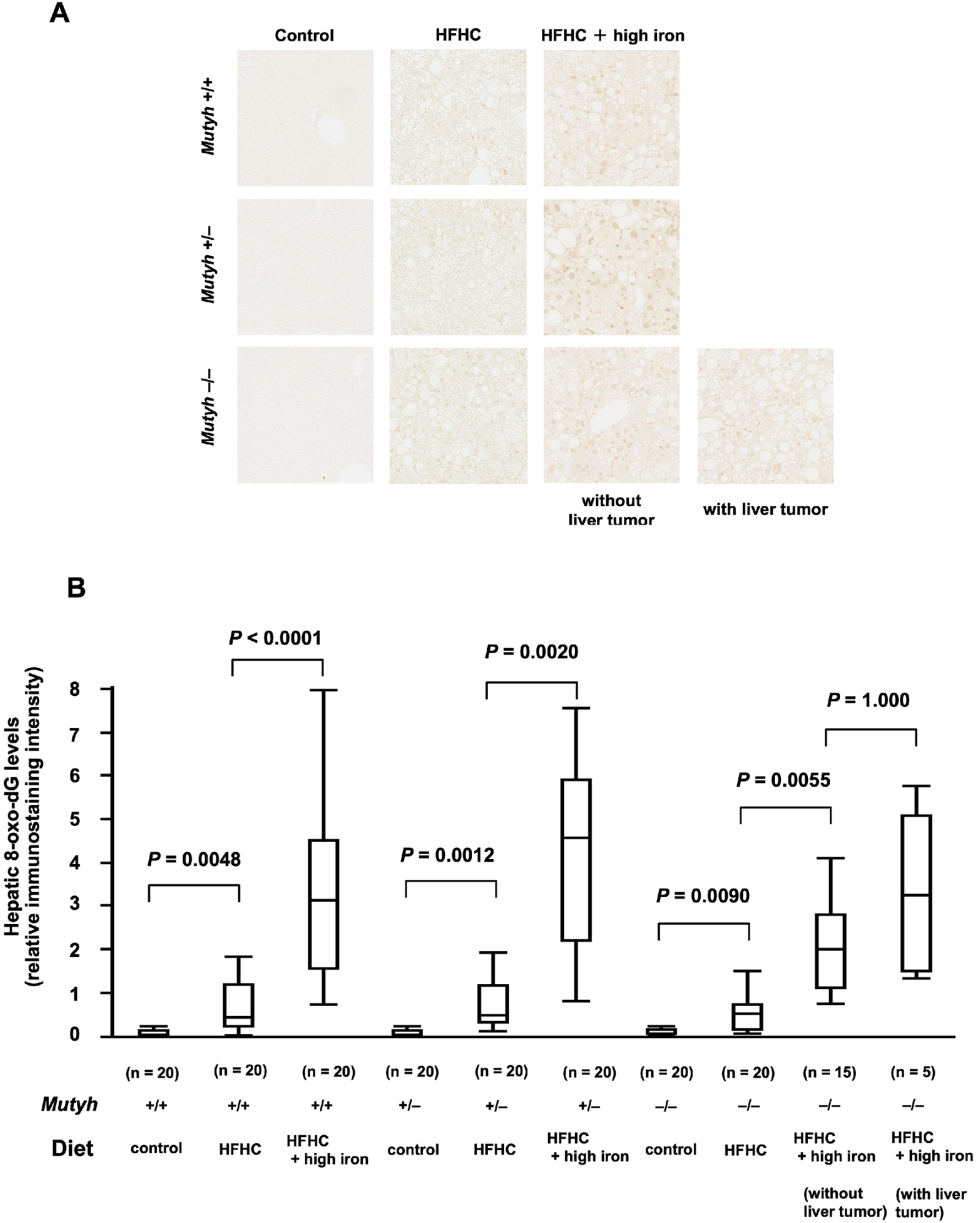
Liver sections stained with anti-8-oxo-dG antibody. (**A**) Immunostaining of mouse livers with anti-8-oxo-dG antibody. (**B**) Semiquantative score of 8-oxo-dG immunostaining. Scores were significantly higher in the HFHC diet groups than the control diet groups (*Mutyh*^+/+^, HFHC diet group vs. *Mutyh*^+/+^, control diet group; *P* = 0.0048, *Mutyh*^+/−^, HFHC diet group vs. *Mutyh*^+/−^, control diet group; *P* = 0.0012, *Mutyh*^−/−^, HFHC diet group vs. *Mutyh*^−/−^, control diet group; *P* = 0.0090) and significantly higher in the HFHC + high-iron diet groups than the HFHC diet groups (*Mutyh*^+/+^, HFHC + high-iron diet group vs. *Mutyh*^+/+^, HFHC diet group; *P* < 0.0001, *Mutyh*^+/−^, HFHC + high-iron diet group vs. *Mutyh*^+/−^, HFHC diet group; *P* = 0.0020, *Mutyh*^−/−^, HFHC + high-iron diet group vs. *Mutyh*^−/−^, HFHC diet group; *P* = 0.0055). There was no significant difference in the index according to the presence or absence of tumor development (*P* = 1.000). Data analysed with a Kruskal–Wallis test followed by Dunn–Bonferroni test. For all data analysis *P* < 0.05 considered significant. Data are shown as box plots for each group of mice. Median values are shown by the line within the box. The bottom and top edges of the boxes represent the 25th and 75th percentiles, respectively. HFHC, high-fat high-carbohydrate; 8-oxo-dG, 8-Hydroxy-2d-deoxyguanosine.

The results of anti-8-oxo-dG immunostaining are shown in Fig. 5A. Comparison of semiquantitative analysis of anti-8-oxo-dG immunostaining showed that the 8-oxo-dG score was are significantly higher in the HFHC diet groups than the control diet groups (*Mutyh*^+/+^, HFHC diet group vs. *Mutyh*^+/+^, control diet group; *P* = 0.0048, *Mutyh*^+/−^, HFHC diet group vs. *Mutyh*^+/−^, control diet group; *P* = 0.0012, *Mutyh*^−/−^, HFHC diet group vs. *Mutyh*^−/−^, control diet group; *P* = 0.0090) and significantly higher in the HFHC + high-iron diet groups than the HFHC diet groups (*Mutyh*^+/+^, HFHC + high-iron diet group vs. *Mutyh*^+/+^, HFHC diet group; *P* < 0.0001, *Mutyh*^+/−^, HFHC + high-iron diet group vs. *Mutyh*^+/−^, HFHC diet group; *P* = 0.0020, *Mutyh*^−/−^, HFHC + high-iron diet group vs. *Mutyh*^−/−^, HFHC diet group; *P* = 0.0055). There was no significant difference in the index according to the presence or absence of tumor development *P* = 1.000) (Fig. 5B).

### Differentially expressed genes and Gene set enrichment analysis

**Figure 6:**
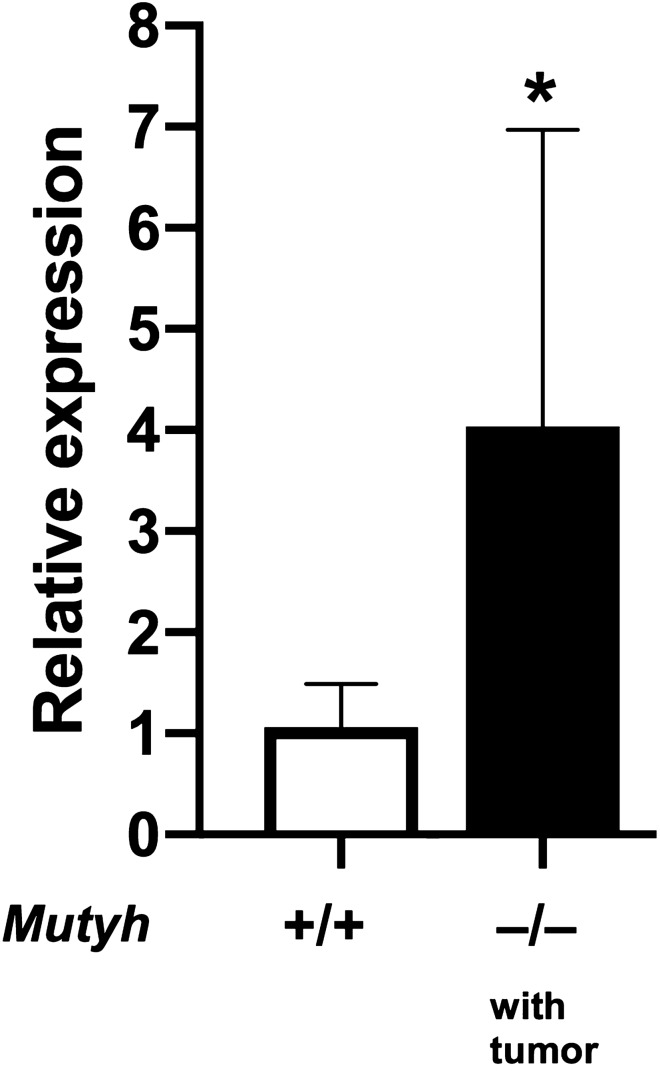
Expression of *Myc* mRNA in non-tumorous hepatic tissue of each groups evaluated by qRT-PCR. Increased *Myc* expression in non-tumorous hepatic tissue of MUTYH-null mice with an HFHC + high-iron diet with liver tumor. Data are the mean of five samples. Error bars represent the SD. * Indicates a significant change (*P* = 0.0318).

To get an insight into the cause of carcinogenesis in the MUTYH-null mice, we used microarray analysis of the *Mutyh*^+/+^ HFHC diet and HFHC + high iron diet groups. We first attempted to identify the DEGs that are highly expressed in MUTYH-null mice with an HFHC + high iron diet (Supplementary Table 2). Unexpectedly, most of the highly expressed genes were immunoglobin-related genes, suggesting that B lymphocytes or plasma cells had infiltrated into the liver tissues of the MUTYH-null mice with an HFHC + high iron diet. Interestingly, *Myc* mRNA was found to be elevated in MUTYH-null mice with an HFHC + high iron diet. We next conducted pathway analysis using GSEA (Table 2). Interestingly, cholesterol homeostasis differed between the wild-type and MUTYH-null mice with an HFHC + high iron diet. Moreover, consistent with the DEGs, genes involved in interleukin (IL)-2/STAT5 signaling was enriched in MUTYH-null mice with liver tumors, indicating that immunological activity in liver could be enhanced in MUTYH-null mice with an HFHC + high iron diet, compared to the wild type. Importantly, the pathway Wnt/β-catenin signaling and apical junctions that correlated with carcinogenesis were enriched in MUTYH-null mice with an HFHC + high iron diet. Remarkably, the *Myc* gene mentioned above is one of the targets of the Wnt/β-catenin signaling pathway. It was confirmed by quantitative reverse transcription-PCR that *Myc* expression was increased in the non-tumorous hepatic tissue of MUTYH-null mice fed an HFHC + high-iron diet and which developed liver tumors (Fig. 6). Collectively, oxidative damage, as well as aberrant Wnt/β-catenin signaling, could disturb cellular functions, such as apical junctions, thereby, carcinogenesis in MUTYH-null mice with an HFHC + high iron diet could develop rather more frequently than in the control group.

**Table 2:** Summary of results of GSEA.

	NES	NOM *P* value	FDR Q-value
Hallmark cholesterol homeostasis	– 1.634014	0.000000	0.144963
Hallmark Wnt/β-catenin signaling	1.387922	0.000000	1.000000
Hallmark apical junction	1.378887	0.000000	1.000000
Hallmark IL-2 STAT5 signaling	1.356143	0.000000	0.906580

GSEA was conducted using the open source software GSEA 3.0. Summary of results of GSEA showing a *P* value < 0.00001 or *Q*-value < 0.25.

FDR, false discovery rate; *GSEA,* gene set enrichment analysis; IL, interleukin; NES, normalized enrichment score; NOM, nominal.

## Discussion

Although it has been shown that oxidative stress is involved in hepatocarcinogenesis in patients with NASH^7,8^, the effects of deficiency of enzymes that repair oxidative DNA damage induced by ROS have not yet been investigated. We examined whether MUTYH deficiency was involved in hepatocarcinogenesis induced by oxidative stress using a NASH model of MUTYH-null mice. Although it has been reported that tumors of the small intestine develop spontaneously in these animals after 18 months, no HCC was observed^22^. We previously reported that development of liver tumors was seen in about one-third of the animals at 12 months in the *Mutyh*^−/−^ high iron diet group. In this study, we demonstrate that liver tumors developed in mice with MUTYH deficiency at 9 months, which is earlier than in mice without an HFHC diet. The incidence of liver tumors was highest in the *Mutyh*^−/−^ HFHC + high iron diet group. Although the incidence was not significant compared to that of *Mutyh*^+/+^ mice, liver tumors developed in a few *Mutyh*^+/–^ mice fed an HFHC diet alone, as well as those fed an HFHC + high iron diet. This may be related to the finding that the livers of mice in the HFHC diet group also had mild, yet more significant, oxidative stress than the mice in the control diet group. The partial MUTYH deficiency may also have been a contributing factor.

To date, there have been many reports on the NASH model mouse but only a few faithfully model the disease process of NASH in humans. Metabolome analysis and transcriptional analysis of these mouse models have shown that the mouse model that most faithfully models NAFLD in humans is the high fat diet (HFD) mouse, although hepatocarcinogenesis has not been observed in these mice^23,24^. Although there have been several reports of murine models in which HCC develops in the liver affected by NASH, the STAM mouse and methionine and choline deficient mouse were considered inappropriate as a model to investigate hepatocarcinogenesis in patients with NASH because these mice showed weight loss due to their abnormal metabolism^25,26^. The NASH model mouse used in the present study seems appropriate for investigating hepatocarcinogenesis induced by decreased MUTYH activity.

Liver tumors do not belong to the characteristic tumor spectrum of MUTYH associated polyposis (MAP) patients. However, there have been reports of the association between MUTYH mutations and liver tumors. Baudhuin et al. compared the percentage of patients with HCC or cholangiocarcinoma who had the MUTYH Y165C and G382D mutations with a non-cancerous control group and reported that there was no significant difference in the frequency of MUTYH mutations between the two groups^18^. Casper et al. investigated the G396D and Y179C mutations, which are the MUTYH hotspot mutations, in Caucasian patients with cholangiocarcinoma and found that the percentage of patients with the mutations was not significantly higher than the control group^19^. On the other hand, Win et al. screened DNA from the first- and second-degree relatives of incident colorectal cancer cases for known MUTYH mutations and found a higher risk of developing liver tumors in those with biallelic and monoallelic MUTYH mutations^20^. We previously investigated the relationships between single nucleotide polymorphisms (SNPs) of genes encoding these enzymes and hepatocarcinogenesis in patients with chronic hepatitis C and identified a SNP of *MUTYH* (minor allele at rs3412958), which reduces *MUTYH* expression, as an independent risk factor for hepatocarcinogenesis^21^. These reports and the findings of this study suggest that MUTYH activity is involved in hepatocarcinogenesis in NASH patients. Further investigation, based on large-scale cohort studies, is required to confirm these findings.

The percentage of positive iron staining in NAFLD cases was reported as 47% by Eder et al., 48% by Hoki et al*.*, 41% by Keith et al*.*, and 68% by Sumida et al.^9,27–29^. These amounts of staining do not represent the severe iron deposition as seen in hemochromatosis but the mild or moderate degrees of hepatic iron accumulation that are common in NAFLD patients^9,22,23^. To overcome the difference between this NASH model mouse and NASH with hepatic iron accumulation in humans, we also examined hepatocarcinogenesis in a model based on feeding a high iron diet to mice on an HFHC diet, so as to induce mild or moderate degrees of hepatic iron accumulation. The amount of iron in the diet was adjusted to achieve mild or moderate degrees of hepatic iron accumulation, as seen in chronic liver diseases according to a previous report^30^. MUTYH deficiency and high iron diet alone required 12 months for hepatocarcinogenesis to develop after the start of intervention^21^; however, we observed the development of liver tumors in a quarter of NASH model *Mutyh*^−/−^ mice, associated with excessive iron in the liver. These results raise the possibility that excessive iron and MUTYH deficiency could be risk factors even for hepatocarcinogenesis in patients with NASH with hepatic iron accumulation.

In the present study, microarray analysis was carried out on non-tumorous liver tissues from MUTYH-null mice with the development of liver tumors and from wild-type mice without the development of liver tumors. GSEA analysis identified the Wnt/β-catenin signaling pathway and *Myc*, a target gene for Wnt signaling, as one of the DEGs, suggesting a constitutive activation of this pathway. The *CTNNB1* gene mutation has been reported in 20–30% of HCC^31,32^. It is noteworthy that *Ctnnb1* mutations were significantly increased in intestinal tumors developed in MUTYH-null mice, suggesting that Wnt/β-catenin signaling pathway is a common target in tumors induced by MUTYH deficiency^33^. We also found a different cholesterol metabolism, suggesting that lipid metabolism could be altered in MUTYH-null mice, although further investigation is required to elucidate the relationship between ROS production and liver dysfunction.

In conclusion, we used a mouse model to demonstrate that decreased MUTYH activity may be involved in hepatocarcinogenesis in some patients with NASH. However, not only the role of monoallelic MUTYH variants, but also the role of other repair enzymes for oxidative DNA damage in hepatocarcinogenesis in NASH patients with iron overload must be evaluated in large cohorts.

## Methods

### Mice

*Mutyh*^+/−^ mice were established as previously described^22^. The *Mutyh*^+/−^ mice were crossbred with C57BL/6 mice for at least 10 generations, then inbred to establish wild-type (*Mutyh*^+/+^), heterozygous (*Mutyh*^+/−^), and MUTYH-null (*Mutyh*^−/−^) mice. All animals were generated and kept in a specific pathogen free (SPF) area. Handling and sacrifice of all animals were done according to the nationally—prescribed guidelines and ethical approval for the studies was granted by the Animal Care and Use Committee of Sapporo Medical University (number of ethics approval 17-053).

### Dietary interventions

The male 8 weeks old mice were fed three types of diet, control diet, high-fat high-carbohydrate (HFHC) diet, and HFHC + high iron diet. The HFHC diet comprises Surwit diet (D12330, Research Diets Inc. New Brunswick, NJ) plus water containing 55% fructose and 45% sucrose^34^. The amount of iron given was 45 mg/kg (D12041503, Research Diets Inc. New Brunswick, NJ) in the control diet, whereas it was 225 mg/kg diet in the high iron diet^30^. In addition, N-acetyl L-cysteine (NAC) (0.2 mg/kg diet) was given to mice in the groups showing a significantly higher incidence of liver tumors.

### Sacrifice, blood tests and tumor development

All mice were sacrificed at 9 months after dietary intervention. Venous blood was taken immediately after sacrifice. Complete blood counts and alanine aminotransferase (ALT) levels were measured at Kishimoto Clinical Laboratory, Inc. (Sapporo, Japan). At the time of sacrifice, all organs, including the liver, gastrointestinal tract (stomach, duodenum, small intestine, and colorectum), kidneys, lungs, and heart were carefully inspected macroscopically for the development of tumors. The liver was fixed in 4% formaldehyde.

### Measurements of hepatic iron concentration

Hepatic iron concentrations were measured as described previously^14,35^. For sample preparation, tissue was dried at 120 °C for 24 h, homogenized and digested using nitric acid and sulphuric acid (1.0 mL of 1 N HNO_3_ and 1.0 mL of 1 NH_2_SO_4_ per 0.1 g dry tissue) while heating. After dilution with de-ionized water (9.0 mL de-ionized water per 1.0 mL of 1 N HNO_3_) and centrifugation at 3000 rpm for 10 min, iron levels were determined using an atomic absorption spectrometer (AAnalyst 800, Perkin Elmer, Norwalk, CT).

### Liver histology and immunostaining

The formalin-fixed liver tissues were stained with hematoxylin & eosin (H&E), Masson trichrome (MT) stains and Berlin blue stain. In addition, the formalin-fixed paraffin embedded specimens were stained (as defined in the manufacturer’s staining protocol) on a BOND-MAX fully automated staining system (Leica Microsystems GmbH, Germany), using 0.5 µg/mL anti-4-hydroxynonenal (4-HNE) monoclonal antibody (Japan Institute for the Control of Aging, Fukuroi, Japan) and anti-8-Hydroxy-2′-deoxyguanosine (8-oxo-dG) antibody (Japan Institute for the Control of Aging, Fukuroi, Japan). Universal negative control for IR-series mouse primary antibody (Dako, Glostrup, Denmark) was used as the negative control.

### Scores for grading and staging of NASH

Liver sections were scored for steatosis grade, lobular inflammation, and fibrosis stage as described previously^32,36^. The steatosis grade was scored from 0 to 3; 0 (none), 1 (< 33% of the lobes), 2 (33–66% of the lobes), and 3 (> 66% of the lobes). Lobular inflammation was scored from 0 to 3; 0 (none), 1 (< 2 foci / field × 200), 2 (2–4 foci / field × 200), and 3 (≥ 5 foci / field × 200). Fibrosis stage was scored from 0 to 4; 0 (none), 1 (zone 3 perivenular perisinusoidal/pericellular fibrosis), 2 (stage 1 and focal or extensive periportal fibrosis), 3 (bridging fibrosis), and 4 (cirrhosis).

### Semi-quantitative assessment of 4-HNE immunostaining

The intensity of 4-HNE immunostaining was scored from 0 to 3, as previously described^7,37^; 0 no staining, 1 mild (punctuated labeling), 2 moderate (dense labeling in a few cells), and 3 strong (dense and homogenous labeling in numerous cells). At least five random fields were examined for each sample and the average of the scores was determined as the 4-HNE index.

### Semi-quantitative assessment of 8-oxo-dG immunostaining

Semi-quantitative assessment of 8-oxo-dG immunostaining was performed as described previously^6,14^. In brief, immunohistochemical analysis of formalin-fixed, paraffin-embedded tissue samples was performed using an avidin–biotin–peroxidase complex technique after microwave antigen retrieval. Sections (4 µm) were successively treated with blocking solution, 1 µg/mL anti-8-oxo-dG monoclonal antibody or normal mouse IgG (Dako, Glostrup, Denmark), biotinylated secondary antibody, and a peroxidase–avidin complex (Envision Plus kit; Dako Japan Co. Ltd, Kyoto, Japan). The intensity of 8-oxo-dG immunostaining in the sections was assessed using an AxioCam photomicroscope and KS-400 image analyzing system (Carl Zeiss Vision GmbH, Hallbermoss, Germany). A microscopic image of each liver section was imported into the KS-400 and brown-stained tissues, which represented positively-stained nuclei of hepatocytes corresponding to 8-oxo-dG immunoreactivity, were converted into a 255-graded gray scale. The average gray scale intensity of each sample was calculated using the KS-400 image analysis program and was represented as the ratio relative to each sequential section immunostained by normal mouse IgG. At least three periportal and three perivenous zones were examined in each section and the average of the scores was determined.

### Microarray analysis

In an attempt to examine the gene expression patterns associated with hepatocarcinogenesis in our model, microarray analysis was carried out on the non-tumorous hepatic tissues from three mice each in the *Mutyh*^+/+^ HFHC + high iron diet group and the *Mutyh*^−/−^ HFHC + high iron diet group. Total RNA was extracted from murine liver tissues using RNeasy Lipid Tissue Mini Kits (QIAGEN, Hilden, Germany), according to the manufacturer’s protocol. After quality check of total RNA, transcriptome analysis was conducted using a GeneChip Human Transcriptome Array 2.0 (HTA 2.0, Affymetrix, Santa Clara, CA) in the GeneticLab Co. (Sapporo, Japan), following the guidelines from Affymetrix. Microarray data have been deposited in the Gene Expression Omnibus under accession number GSE157142.

### Analysis of differentially expressed genes and pathway analysis

Analysis of differentially expressed genes and pathway analysis were performed as described previously^38^. In brief, summation and normalization of probe set expression measures was carried out using the Robust Multi-Array Average (RMA) provided from analysis by Bioconductor (R commander 3.6.1). Each dataset was saved as a matrix in text format and each dataset was reloaded into RStudio (version1.2.1335) using the read.table function. To detect differentially expressed genes (DEGs) between the *Mutyh*^+/+^ HFHC + high iron diet (n = 3) and the *Mutyh*^−/−^ HFHC + high iron (n = 3) groups, the limma package was used after RMA normalization (supplementary Table 1). Gene set enrichment analysis (GSEA) was conducted using the open source software GSEA 3.0 (http://software.broadinstitute.org). For gene sets databases, h.all.v6.0.symbol.gmt [Hallmarks], c2.cp.biocarta.v6.0.symbols.gmt [Curated], c2.cp.kegg.v6.0.symbols.gmt and c2.cp.reactome.v6.0.symbols.gmt [Curated] were used. The pathways showing nominal (NOM) *P* value < 0.05 or false discovery rate (FDR) *Q*-value < 0.25 were considered to be significant.

### RNA extraction and quantitative reverse transcription-PCR

RNA was extracted with TRIZOL Reagent (Thermo Fisher Scientific, Waltham, MA, USA) in accordance with the manufacturer's protocol. Total RNA (1 μg) was reverse-transcribed by using a SuperScript VILO cDNA Synthesis kit (Thermo Fisher Scientific, Waltham, MA, USA), and q-PCR was performed with an Applied Biosystems 7300 Real-time PCR system (Applied Biosystems, Foster City, CA, USA). Analysis of target genes was conducted in quadruplicate using the POWER SYBR Green Master Mix (Thermo Fisher Scientific, Waltham, MA, USA) as previously described^39^. Transcripts levels were normalized to *β-*actin expression. The experiments were repeated three times. The sequences of the PCR primer are shown in Supplementary Table 3.

### Statistical analysis

All statistical analyses were performed using JMP 13.1.0 software (SAS, Cary, NC). Data from murine blood tests, semi-quantitative scores of immunohistochemical staining in *Mutyh*^+/+^ and *Mutyh*^+/−^ groups were compared using the Mann–Whitney U test. Histological findings of liver sections (steatosis grade, lobular inflammation, and fibrosis stage) and semi-quantitative scores of immunohistochemical staining in *Mutyh*^−/−^ group were analyzed Kruskal–Wallis test followed by Dunn–Bonferroni test. The incidence of liver tumors was compared using chi-square test. *Myc* expressions were analyzed using unpaired t test. Furthermore, all statistical tests were two-sided, and *P* < 0.05 was considered statistically significant. All authors had access to the study data and have reviewed and approved the final manuscript.

## Supplementary Information

Supplementary Tables

## Abbreviations

ALT: Alanine aminotransferase
HCC: Hepatocellular carcinoma
HCV: Hepatitis C virus
HFHC: High-fat high-cholesterol
NAC: N-acetyl L-cysteine
NAFLD: Non-alcoholic fatty liver disease
NASH: Non-alcoholic steatohepatitis
ROS: Reactive oxygen species
SNP: Single nucleotide polymorphism
4-HNE: 4-Hydroxynonenal
8-oxo-dG: 8-Hydroxy-2′-deoxyguanosine
