# Supplementary material for: MUTYH is associated with hepatocarcinogenesis in a non-alcoholic steatohepatitis mouse model
Source: Sci Rep. 2021 Feb 11;11:3599. doi: 10.1038/s41598-021-83138-8 (PMC7878918; doi:10.1038/s41598-021-83138-8)
Supplement: Supplementary file 1 — Supplementary Tables [file 41598_2021_83138_MOESM1_ESM.pdf]

MUTYH is associated with hepatocarcinogenesis in a non-alcoholic steatohepatitis mouse model

Hiroki Sakamoto[1]· Koji Miyanishi*[1]· Shingo Tanaka[1]· Ryo Ito[1]· Kota Hamaguchi[1]· Akira Sakurada[1]· Masanori Sato[1]· Tomohiro Kubo[1]· Takahiro Osuga[1]· Kazuyuki Murase[1]· Kohichi Takada[1]· Yusaku Nakabeppu[3]· Masayoshi Kobune[2]· Junji Kato[1]

[1] Department of Medical Oncology, Sapporo Medical University School of Medicine, South-1, West-16, Chuo-ku, Sapporo, 060-8543, Japan

[2] Department of Hematology, Sapporo Medical University School of Medicine, South-1, West-16, Chuo-ku, Sapporo, 060-8543, Japan

[3] Division of Neurofunctional Genomics, Department of Immunobiology and Neuroscience, Medical Institute of Bioregulation, Kyushu University, 3-1-1 Maidashi, Higashi-Ku, Fukuoka 812-8582, Japan

Suppl. Table 1. Histological findings of liver sections.

	Control diet			HFHC diet			HFHC + high-iron diet		
Mutyh	+/+	+/−	−/−	+/+	+/−	−/−	+/+	+/−	−/−
n	20	20	20	20	20	20	20	20	20
Steatosis grade (0-3)	0.08 ± 0.08	0.10 ± 0.07	0.12 ± 0.08	1.90 ± 0.10	2.06 ± 0.15	1.84 ± 0.16	2.00 ± 0.15	1.90 ± 0.16	1.95 ± 0.17
Lobular inflammation (0-3)	0.15 ± 0.10	0.26 ± 0.10	0.29 ± 0.11	0.65 ± 0.11	0.67 ± 0.11	0.70 ± 0.15	0.65 ± 0.13	0.70 ± 0.11	0.65 ± 0.11
Fibrosis stage (0-4)	0.00 ± 0.00	0.05 ± 0.05	0.11 ± 0.08	0.65 ± 0.15	0.72 ± 0.14	0.68 ± 0.19	0.70 ± 0.13	0.75 ± 0.12	0.75 ± 0.16

Data expressed as the mean ± SEM.

There were no significant differences in steatosis grade, lobular inflammation, and fibrosis stage among the Mutyh genotypes in the control diet group. In the HFHC diet group and HFHC + high-iron diet group, the results were similar.

In *Mutyh*$^{+/+}$ mice, the steatosis grade of the HFHC diet group and HFHC + high-iron diet group was significantly higher than that of the control diet group. The results were similar for lobular inflammation and fibrosis stage. (Steatosis grade: control diet group vs. HFHC diet group; $P < 0.0001$, control diet group vs. HFHC + high-iron diet group; $P < 0.0001$)(Lobular inflammation: control diet group vs. HFHC diet group; $P = 0.0228$, control diet group vs. HFHC + high-iron diet group; $P = 0.0333$)(Fibrosis stage: control diet group vs. HFHC diet group; $P = 0.0063$, control diet group vs. HFHC + high-iron diet group; $P = 0.0016$)

In *Mutyh*$^{+/-}$ mice, the steatosis grade of the HFHC diet group and HFHC + high-iron diet group was significantly higher than that of the control diet group. The results were similar for lobular inflammation and fibrosis stage. (Steatosis grade: control diet group vs. HFHC diet group; $P < 0.0001$, control diet group vs. HFHC + high-iron diet group; $P < 0.0001$)(Lobular inflammation: control diet group vs. HFHC diet group; $P = 0.0453$, control diet group vs. HFHC + high-iron diet group; $P = 0.0207$)(Fibrosis stage: control diet group vs. HFHC diet group; $P = 0.0007$, control diet group vs. HFHC + high-iron diet group; $P = 0.0002$)

In *Mutyh*$^{-/-}$ mice, the steatosis grade of the HFHC diet group and HFHC + high-iron diet group was significantly higher than that of the control diet group. The results were similar for lobular inflammation and fibrosis stage. (Steatosis grade: control diet group vs. HFHC diet group; $P < 0.0001$, control diet group vs. HFHC + high-iron diet group; $P < 0.0001$)(Lobular inflammation: control diet group vs. HFHC diet group; $P = 0.0209$, control diet group vs. HFHC + high-iron diet group; $P = 0.0213$)(Fibrosis stage: control diet group vs. HFHC diet group; $P = 0.0259$, control diet group vs. HFHC + high-iron diet group; $P = 0.0045$)

Data analysed with a Kruscal-Wallis test followed by Dunn-Bonferroni test.

HFHC, high-fat high-carbohydrate

Suppl. Table 2. DEGs in the livers of Wild type vs. MutYH KO mice by limma

	Gene Symbol	logFC	AveExpr	t	P.Value	adj.P.Val	B
1	Ighv1-72	-1.676128	6.661375	-2.955926	0.018082	0.981233	-3.708059
2	Igkv4-72	-1.421596	5.945208	-2.775994	0.023869	0.981233	-3.788456
3	LOC382693	-1.158520	2.410206	-2.655847	0.028769	0.981233	-3.844152
4	LOC102642252	-1.065014	5.803227	-3.016090	0.016489	0.981233	-3.682015
5	Myc	-0.969419	6.582124	-2.671121	0.028093	0.981233	-3.836986
6	Cish	-0.964983	5.547243	-3.983437	0.003970	0.981233	-3.322539
7	Orm3	-0.960184	7.135387	-4.274395	0.002653	0.981233	-3.235183
8	Igkv16-104	-0.895566	4.315239	-3.553842	0.007361	0.981233	-3.468534
9	Foxq1	-0.883993	6.776197	-3.110517	0.014277	0.981233	-3.642007
10	Igk	-0.775381	3.779171	-2.663833	0.028413	0.981233	-3.840402
11	Gbp11	-0.757120	5.827069	-2.792987	0.023248	0.981233	-3.780706
12	5031426D15Rik	-0.745124	3.053524	-3.799466	0.005155	0.981233	-3.382485
13	Gadd45g	-0.731322	6.811322	-2.572363	0.032770	0.981233	-3.883745
14	Mir5128	-0.719123	3.961921	-5.144180	0.000857	0.981233	-3.021770
15	Ms4a4a	-0.717654	4.703872	-4.399909	0.002239	0.981233	-3.200168
16	Gm16348	-0.712945	6.812482	-7.190625	0.000089	0.981233	-2.714914
17	Phlda1	-0.710061	7.096436	-2.555996	0.033619	0.981233	-3.891590
18	LOC238440	-0.705779	1.721518	-6.173361	0.000258	0.981233	-2.841001
19	Rhox2f	-0.702325	2.068572	-3.999797	0.003880	0.981233	-3.317389
20	Trgv2	-0.683663	4.313274	-2.608243	0.030985	0.981233	-3.866642
21	Igfbp2	-0.683538	8.062563	-2.821109	0.022258	0.981233	-3.767951
22	Ighv1-61	-0.680564	2.068829	-3.342208	0.010069	0.981233	-3.548391
23	Gm15293	-0.671304	2.735273	-3.221855	0.012062	0.981233	-3.596211
24	Atp6v0d2	-0.665701	5.141780	-3.761672	0.005443	0.981233	-3.395271
25	Snord91a	-0.658174	4.109664	-3.293289	0.010834	0.981233	-3.567616
26	Ugt2b37	-0.643084	3.184682	-2.676179	0.027872	0.981233	-3.834618
27	Vmn1r103	-0.630419	3.069672	-2.863596	0.020844	0.981233	-3.748850
28	Gm14459	-0.630259	1.903076	-4.022304	0.003760	0.981233	-3.310351
29	Ttc39aos1	-0.615441	8.645148	-5.654103	0.000464	0.981233	-2.924101
30	Olfr1031	-0.615230	2.620036	-3.605512	0.006825	0.981233	-3.449847

Log FC indicated Log_2 (Wild type / MutYH KO)

Suppl. Table 3. The primer pairs used for qPCR analysis.

The primer pairs used for qPCR analysis of the gene signature in murine samples are reported in the following table.
The nucleotide sequence of Myc was chosen using the geNorm software (https://www.ncbi.nlm.nih.gov/gene/).
β-actin was chosen as a reference gene using NCBI Gene ID (http://medgen.ugent.be/~jvdesomp/genorm/).
Gene expression values were obtained by applying the Delta Ct method.

Target		Product size, bp	Sequence (5'.3')	PCR conditions (Tm, °C)	Sample origin
MYC	Forward	151	AGCCCCTAGTGCTGCATGAG	60.4	murine
	Reverse	151	CCTCGGGATGGAGATGAGC	60.4	murine
β-actin	Forward	245	GTGACGTTGACATCCGTAAAGA	56.7	murine
	Reverse	245	GCCGGACTCATCGTACTCC	60.4	murine
